# Gentamicin-Loaded Chitosan Nanoparticles Improve Its Therapeutic Effects on *Brucella*-Infected J774A.1 Murine Cells

**DOI:** 10.31661/gmj.v8i0.1296

**Published:** 2019-10-29

**Authors:** Ali Razei, Abdol Majid Cheraghali, Mojtaba Saadati, Mahdi Fasihi Ramandi, Yunes Panahi, Abbas Hajizade, Seyed Davar Siadat, Ava Behrouzi

**Affiliations:** ^1^Applied Biotechnology Research Center, Baqiyatallah University of Medical Sciences, Tehran, Iran; ^2^Faculty of Pharmacy, Baqiyatallah University of Medical Sciences, Tehran, Iran; ^3^Biology Research Centre, Faculty of Basic Sciences, Imam Hossain University, Tehran, Iran; ^4^Molecular Biology Research Center, Baqiyatallah University of Medical Sciences, Tehran, Iran; ^5^Chemical Injuries Research Center, Baqiyatallah University of Medical Sciences, Tehran, Iran; ^6^Pasteur Institute of Iran (IPI), Mycobacteriology and Pulmonary Research Tehran, Iran

**Keywords:** *Brucella melitennis*, *Brucella abortus*, Chitosan, Gentamicin, Nanoparticles

## Abstract

**Background:**

Final elimination of some intracellular bacterial agents, such as Brucella, is often a complex issue and impossible to achieve, primarily due to the presence and survival of the bacteria within phagocytic cells. By penetrating into the cell membrane, drug delivery nanosystems can reduce the number of intracellular bacteria. The aim of this study was to assess the efficacy of chitosan nanoparticles on the delivery of gentamicin into *Brucella* infected J774A.1 murine cells in vitro.

**Materials and Methods:**

Chitosan nanoparticles (NPs) were synthesized using ionic gelation technique. The shape, size and charge of NPs, loading rate and release of the drug were investigated. Finally, the effects of gentamicin-loaded chitosan NPs (Gen-Cs) and free gentamicin on J774A.1 murine cells infected with these bacteria were examined.

**Results:**

The mean size and charge of NPs were computed as 100 nm and +28mV, respectively. The loading capacity of NPs was 22%. About 70% of the drug was released from NPs during the first 8 hours. Antimicrobial activity of the two formulations showed that MIC (minimum inhibitory concentration) of the Gen-Cs and free drug was 3.1 and 6.25 µg, respectively. The minimum bactericidal concentration of the NPs-loaded drug and free drug was 6.25 and 12.5 µg, respectively. Cell culture analysis revealed that there was a significant reduction in the load of the intercellular bacteria in J774A.1 murine cells in both formulations.

**Conclusion:**

Our results showed the Gen-Cs have a proper potential for optimal treatment of intracellular bacterial agents.

## Introduction

Brucellosis is a prevalent zoonotic disease caused by the genus *Brucella melitensis.* More than 500,000 people are annually infected with the disease and it is estimated that 2.4 billion people are at risk [1]. Since the bacteria reside inside the macrophages, it is practically difficult to treat the disease and sometimes, it is associated with relapse [2]. The World Health Organization (WHO) recommends the administration of two antibiotics, including doxycycline and rifampin for 6 months for the treatment of the disease [3, 4]. However, recently, it has been reported that the administration of doxycycline antibiotic in combination with streptomycin or gentamicin is efficacious in combating the disease [5]. However, despite the direct health effects, most of these antibiotics are virtually ineffective in the complete elimination of the bacteria and a relapse rate of 5% to 10% in humans has been reported [6].

Aminoglycosides are considered as effective antimicrobial agents, especially against Gram-negative and anaerobic bacilli. Among them, gentamicin is an antibiotic with a wide range of antimicrobial properties and exhibits much more efficacy than streptomycin against *Brucella* in clinical samples [7]. The expression and induction of some *Brucella virulence* genes are inhibited by this antibiotic. These properties stimulate gentamicin to act as a suitable drug in the treatment of brucellosis [8]. However, the high water solubility of gentamicin reduces its penetration into the cells and, consequently, the treatment of intracellular bacterial infections using this antibiotic poses a serious challenge [9-11].

These problems may be solved employing drug delivery systems and such diseases can now be cured more efficiently than it was previously [12-15].

Nowadays, nanotechnology has created hope and opportunity for the treatment of intracellular infections using nanoparticles (NPs) as drug carriers as well as surmount the above-mentioned problems or, at least, enhance and optimize therapeutic effects of drugs when compared to the free ones [16, 17]. These systems can lead to improvement in therapies, reduce the accumulation of drug inside the cell, as well as reduce the required doses of drug and the number of times of administration [18, 19].

On the other hand, research have demonstrated that NPs are able to forestall the degradation of drugs and control their delivery and encapsulation [20].Therefore developing a targeted and controlled delivery system may lead to optimization of the drug and its bioavailability in the cells, culminating in a reduction in its side effects [21]. In the present study, the potential of gentamicin-loaded chitosan NPs (Gen-Cs) as a new formulation against two *Brucella* strains: *B. melitensis* and *B. abortus* was investigated.

## Materials and Methods

*B. melitensis* ATCC23457, *B. abortus* S19 ATCC23448 and J774A.1 murine macrophage-like cells were purchased from Pasteur Institute of Iran (Tehran, Iran). *B. melitensis* and *B. abortus* S19 were cultured on *Brucella* agar at 37°C with 5% CO2. J774A.1 murine macrophage-like cells were exploited in order to carry out cell culture studies. To this end, the cells were grown in Dulbecco’s Modified Eagle’s Medium (DMEM) supplemented with 1% penicillin-streptomycin (Cellgro, USA) and 10% heat-inactivated fetal bovine serum (FBS) in a humidified atmosphere of 5% CO2 at 37°C. A 140 kDa chitosan molecules, with deacetylation degree of 85%, tripolyphosphate (TPP) and glacial acetic acid were purchased from Merck (Germany) and Sigma (USA), respectivelty. Gentamicin was purchased from Biobasic (Ontario, Canada).

### Preparation of Chitosan NPs

The ionic gelation process was employed to prepare the chitosan NPs by using a previously described technique [22]. The chitosan molecules were briefly dissolved in 300µl acetic acid of 0.2% (w/v). Then, the volume increased to 30 ml by addition of sterile double water. The mixture was stirred overnight employing a magnetic stirrer at laboratory temperature and a speed of 400 rpm. The TPP was added dropwise to the mixture at a 3:1 chitosan/TPP ratio. The generated NPs were stirred employing magnetic stirrer for 1 h. The solution was then centrifuged for 30 min at 18000 rpm. The supernatant was discarded and the NPs, which settled at the tube, were lyophilized and stored at -20ᵒC [23].

### Properties of the Gen-Cs

Scanning electron microscopy (SEM) imaging (JEOL 7500 F) was used to analyze the surface morphology. Additionally, the size of NPs Zetasizer (Malvern Instruments, Malvern, UK) was exploited to measure the NPs’ζ potential.

### Determination of the loading efficiency of drug onto chitosan NPs

In order to determine the loading efficiency of the drug onto NPs, the drug-loaded NPs solution was centrifuged at 16000 rpm for 30 minutes. Then, the absorption of the supernatant was read at a wavelength of 334 nm with an ELISA reader in OPA solution (o-phtaldialdehyde). A solution containing only the NPs was used as the blank. Afterwards, the loading and entrapment efficiency were determined, which was followed by drawing of standard curve for the drug concentration.[24] Entrapment efficiency (EE) and loading capacity (LC) were obtained employing Equation 1 and 2, respectively.

Equation 1: EE=F/T×100

Equation 2: LC=F/W ×100

where F is the free drug concentration in the supernatant, T is the total concentration of drug and W is the weight of NPs.

### Determination of Drug Desorption from NPs

To examine the release profile of the drug from NPs, NPs carrying a specific concentration of drug were re-suspended in 2 ml of PBS (pH 7.4) and checked within 48 hours. At the end, the absorbance of all samples were read at 334 nm wavelength using an ELISA reader.

### Evaluation of the Cytotoxicity of NPs

To evaluate the toxicity effects of NPs on the J774A.1 murine cells, MTT assay was performed. Different concentrations of Gen-Cs in the range of 0 to 480 µg were prepared momentarily. Then, the cells were cultured in a 75 cm2 flasks and incubated at 37ᵒC. After 24 h, the cells were trypsinized and centrifuged at 1100 g for 5 min. The pellet was re-suspended in a fresh media such that the final number of cells in each well was 105. Then, different concentrations of NPs were added to the wells and the plate was incubated in a CO2 incubator with 5% CO2 at 37°C for two different time periods, 24 and 48 h. Thereafter, (3-[4,5-dimethylthiazole-2-yl]-2,5-diphenyltetrazolium bromide) was added to each well and the plates were again incubated at the previous conditions for 4 h. After this time, MTT solution was removed and in order to dissolve the formazan crystals, 50 µl of DMSO was added to each well and the absorbance was read at 540 nm employing an ELISA reader (Bio-Rad Laboratories, USA). The survival rate was computed using Equation 3.

Equation 3: AT/AC × 100

Where AT and AC represent the absorbance of treated and control cells, respectively. Heather and Feix protocol was exploited for MTT assay of J744A.1 murine cells [25].

### Study of the Antimicrobial Effects of the Formulations

To study the antimicrobial properties of the formulations, turbidimetric method was employed. [26]. To this end, first, Gen-Cs and free drug were sterilized employing UV radiation for 30 minutes. Then, a certain concentration of bacteria equivalent to 0.5 McFarland standard was prepared in Luria Bertani medium. Thereafter, the effects of the three formulations, including bare chitosan NPs, free gentamicin (Gen) and chitosan NPs containing gentamicin (Gen-Cs) were applied. First, formulation, i.e.bare NPs were used as the control. Gen-Cs and Gen were added to wells in the final concentrations ranging from 0 to 50µg/ml. The plate was incubated at 37°C for 18 to 24 h. Subsequently, tubes were assessed for turbidity. The well with no turbidity was considered as minimum inhibitory concentration (MIC). To obtain the minimum bactericidal concentration (MBC), the mixtures of wells containing higher concentrations of drug (in comparison to MIC) were added to a plate containing *Brucella* agar and the results were seen after 24 h of incubation at 37°C.

### In Vitro Infection Assay Using Cell Culture

To investigate the in vitro activities of the NPs, 48 h before infection, J774A.1 murine macrophage-like cells were seeded in 24 well plates (at a density of *C*5 × 105 cells per well). Dulbecco’s Modified Eagle’s Medium (DMEM) supplemented with 10% heat-inactivated fetal calf serum (FCS, Sigma Co.) was used to grow the cells. Plates were kept in a humidified 5% CO2 atmosphere at 37°C. The macrophage cells were infected with *B. abortus* S19 and *B. melitensis* for 1 h at a 1:100 multiplicity of infection. In order to kill and wash off non-phagocytized bacteria, before addition of the media, the cells were washed with DMEM medium containing 50 μgmL−1 gentamicin (three times). DMEM medium was used for toe cells 24 h after the infection with either free drug (150 μg gentamicin per well) or Gen-Cs (105 μg gentamicin per well). It should be noted that the loading efficiency was computed as 70%; therefore, having a primary concentration of 150 μg gentamicin, the final concentration of gentamicin in NPs, which was 105 μg was added to the infected cells and incubated further for 18 h. Chitosan NPs without antibiotic and non-treated infected cells were considered as control groups. CFUs of *Brucella* were determined in the lysates of the infected cells. To this end, a series of 10-fold serial dilutions of the lysates were plated on TSA and the plate was incubated at 37°C under 5% CO2.

### Statistical analysis

Statistical analyses were performed employing Graph Pad PRISM 6. All experiments were conducted in triplicate. One-way (ANOVA) and post-hoc Tukey’s HSD were used to compare the different groups. P-values smaller than 0.05 were considered statistically significant.

## Results

### Analyzing the morphology, size and zeta potential of NPs

The morphological characteristics and size of drug-loaded NPs were evaluated using SEM imaging. As shown in Figure-2, the drug-loaded nanoparticles had spherical shapes and size distribution analysis showed that the NPs had an average size of 100 nm with minimum size of 70 nm and maximum size of 150 nm (Figure-2). Analysis of the NPs’ zeta potential using the zetasizer revealed that the NPs had a zeta potential of +28 mv. The loading capacity and loading efficiency of the system was computed as22 and 72%, respectively.

### Investigation of drug release from chitosan NPs

Study of the drug release profile showed that a relatively burst release occurred during the initial 8 hours, such that about 70% of the drug was released during this time. However, after 8 hours, the drug release reached a steady state and as can be seen in Figure-3, after 48 hours, about 15% of the drug had not been released from the NPs. Release profile was investigated employing four models, including zero-order, first-order, Higuchi and Power Low with function models of, ,, and, respectively. As can be seen in Figure-3, the release profile was best-fitted for the first-order model with an R2 of 0.90.

### MTT Assay

To investigate the cytotoxicity effect of the NPs, MTT assay was performed. As can be seen in Figure-4, after 24 h, no significant cytotoxicity was observed at any applied concentration. Since the applied concentration of Gen-Cs in the treatment of the infection was lower than these concentrations, it was concluded that the applied NPs had no significant cytotoxicity (P-value>0.05).

### Investigation of the antimicrobial properties of NPs

After 24 h incubation at 37°C, following the application of Gen-Cs, MIC was measured as 3.1 µg for both bacterial strains; while for the free gentamicin, the MIC was computed as 6.25 µg. The MBC of the drug-loaded NPs for both bacterial strains was calculated as 6.25 µg, while the MBC of the free drug for both bacteria was measured as 12.5 µg.

### In vitro cell culture assay

Intracellular reduction of *B. melitensis* and *B. abortus* S19 in infected J774.A1 murine cell line was measured and compared in different forms as follows: after incubation with the Gen-Cs, after incubation with free antibiotics, after incubation with bare NPs and after incubation with PBS. The Gen-Cs and free drug were able to efficiently decrease the number of bacteria in the infected cells, while the number of bacteria in cells treated with bare NPs and untreated cells did not reduce significantly.

## Discussion

In the present study, chitosan NPs were exploited to deliver gentamicin into *Brucella* infected macrophages in order to examine the applicability of these NPs as carriers in intracellular drug delivery. The NPs were prepared by ionic gelation technique. The synthesized NPs had an average size of about100 nm. This size is of interest because such particles are uptaken by cells via clathrine-mediated endocytosis [27, 28]. The zeta potential of the prepared NPs was computed as +28 mV, which, again, is a suitable charge for the NPs to be uptaken by the cells due to the negative charge of cell membranes, which makes positively charged NPs to have higher internalization levels. [29]. The loading capacity of gentamicin into chitosan NPs was calculated as 22%. The low loading capacity of chitosan NPs can be attributed to the positive net charge of the antibiotic. Since the net charge of the NPs was positive, the positively charged antibiotic could not be loaded onto the NPs efficiently. There are many lines of evidence that buttress this assertion [23]. Investigation of the release profile showed that there was a burst release during the initial 8 hours and after this time, the release occurred more slowly. The results of the release profile showed that the release pattern was best-fitted for the first-order model, for which it showed that the drug was adsorbed onto the NPs as well as being entrapped in these particles. Investigation of the antimicrobial properties of chitosan NPs containing gentamicin using (minimum inhibitory concentration) MIC and MBC (minimum bactericidal concentration) assays showed that this formulation was effective in the elimination of both *B. melitensis* and *B. abortus* strains. MIC and MBC of nano-formulation for both strains were 3.1 and 6.25 µg, respectively, while the MIC and MBC of the free drug were computed as 6.25 and 12.5µg, respectively. The higher antibacterial activity of nano-formulation in comparison to the free drug was probably due to the sustained release of the drug in the former state; while in the case of the free drug, the duration of the activity was low. In the nano-formulation, the drug was released slowly; hence, the activity was lasted for a long period. Investigation of the effect of the gentamicin-loaded NPs and free gentamicin showed that nano-formulation was more efficient in the reduction of the bacterial load inside the cells; while 150 µg of the free drug reduced the bacterial load down to 103 and 150 µg of gentamicin loaded-NPs was able to reduce the bacterial load to 102. The higher efficiency of the entrapped drug in this regard can be attributed to the ability of the chitosan NPs to penetrate the cells. The prepared chitosan NPs had an average size of 100 nm. As explained before, NPs with this size are efficiently uptaken by cells via endocytosis. It has been shown that free gentamicin is uptaken by cells via pinocytosis, a mechanism that is less efficient than endocytosis in the uptake of foreign materials [30]. Indeed, the bioavailability of gentamicin-loaded chitosan NPs is higher than free gentamicin. In the case of the free drug, the cells were exposed to high concentrations of the drug for a short time, but, in the case of the drug containing NPs, the drug was available for a longer time and therefore, it was able to reduce the bacterial load more efficiently. In a study conducted by Prior *et al*., they compared the efficiency of PLGA microspheres containing gentamicin and free gentamicin in the reduction of *B. abortus* in cells. They observed that the entrapment of the drug in microparticles could reduce the bacterial load more efficiently compared to the free drug [31]. This result is in consonance with our finding. However, slight differences between the results of these two studies may be related to the types of NPs and their sizes. Studying the *in vivo* efficiency of a modified form of gentamicin on the load reduction of *B. melitensis*, Imbuluzqueta *et al*., demonstrated that particulate drug and PLGAencapsulated drug, are more efficient in the eradication of intracellular pathogens than free drug. Many different studies have also shown the promising applicability of different NPs in the delivery of drugs against intracellular pathogens [32].

## Conclusion

Overall, the results of the present study showed the applicability of gentamicin-entrapped chitosan NPs for the treatment of *B. melitensis* and *B. abortus* infected cells. Based on the results, it can be concluded that nanoparticles containing the drug have a great potential to optimize the treatment of intracellular infections

## Acknowledgement

The aforementioned article was done based on the student’s thesis research work at the Applied Biotechnology Research Center, Faculty of Pharmacy and Chemical Injuries Research Center, Baqiyatallah University of Medical Sciences and Pasteur Institute. We appreciate all staff and officials of these centers.

## Conflict of interest

The authors declare that there were noconflict of interest regarding this paper.

**Figure 1:**
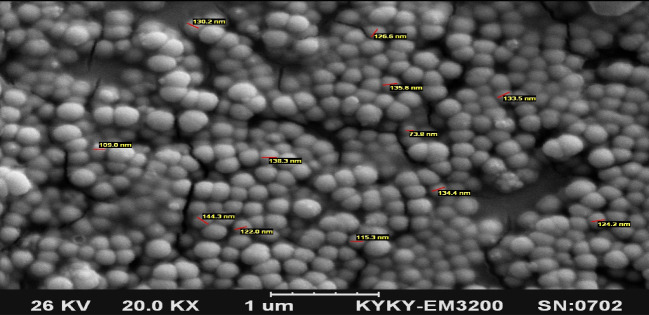
Scanning electron microscopy of drug-loaded NPs.

**Figure 2:**
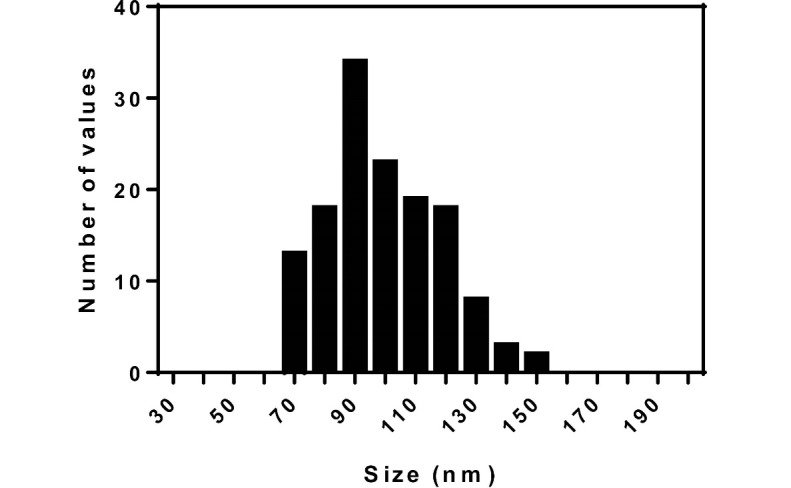
Size distribution of NPs. The graph was depicted using a size of 136 NPs.

**Figure 3:**
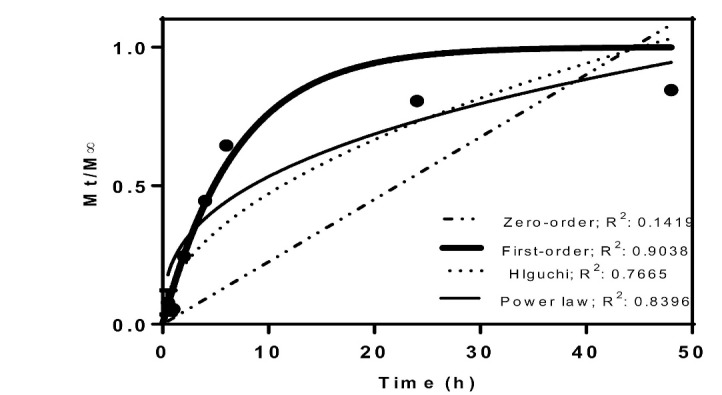
Drug release profile. Four models were evaluated to obtain the best-fitted model. As R2 values showed, the release profile was best-fitted for first-order model.

**Figure 4:**
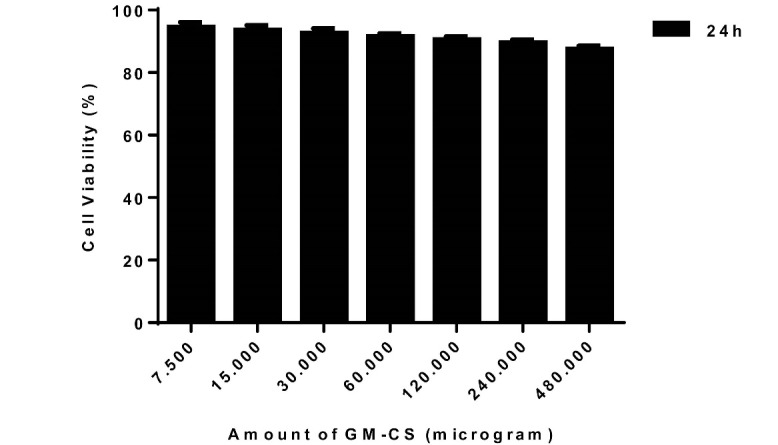
MTT assay for investigation of the cytotoxicityof NPs. No significant cytotoxicity was observed after 24 h at any applied concentrations.

**Figure 5:**
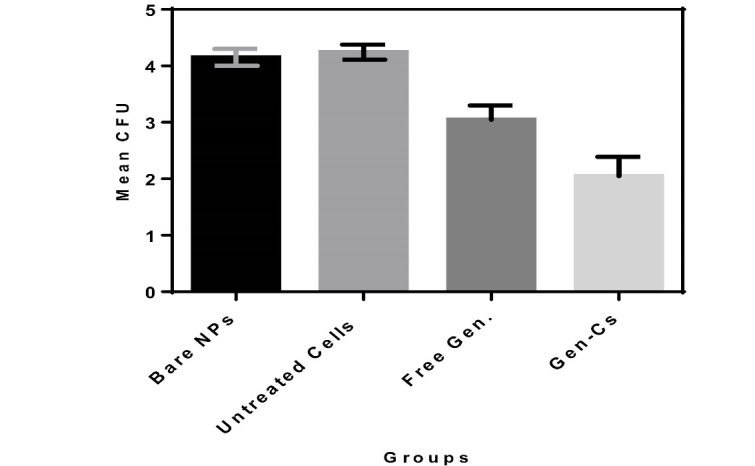
*In vitro* infection assay. Intracellular *B. melitensis* and *B. abortus* S19 reduction in infected J774.A1 macrophage cell line after incubation for 18 h with four different formulations : drug-loaded NPs (Gen-Cs), free gentamicin (Free Gen.), bare NPs and untreated infected cells. It should be noted that the presented data (CFU counts) shown on the graph were obtained with *B. melitensis*. However, similar data were obtained for *B. abortus.*
